# Signaling control of the constitutive androstane receptor (CAR)

**DOI:** 10.1007/s13238-013-0013-0

**Published:** 2014-01-29

**Authors:** Hui Yang, Hongbing Wang

**Affiliations:** Department of Pharmaceutical Sciences, University of Maryland School of Pharmacy, 20 Penn Street, Baltimore, MD 21201 USA

**Keywords:** constitutive androstane receptor, nuclear translocation, phosphorylation, signaling regulation

## Abstract

The constitutive androstane receptor (CAR, NR1I3) plays a crucial role in the regulation of drug metabolism, energy homeostasis, and cancer development through modulating the transcription of its numerous target genes. Different from prototypical nuclear receptors, CAR can be activated by either direct ligand binding or ligand-independent (indirect) mechanisms both initiated with nuclear translocation of CAR from the cytoplasm. In comparison to the well-defined ligand-based activation, indirect activation of CAR appears to be exclusively involved in the nuclear translocation through mechanisms yet to be fully understood. Accumulating evidence reveals that without activation, CAR forms a protein complex in the cytoplasm where it can be functionally affected by multiple signaling pathways. In this review, we discuss recent progresses in our understanding of the signaling regulation of CAR nuclear accumulation and activation. We expect that this review will also provide greater insight into the similarity and difference between the mechanisms of direct vs. indirect human CAR activation.

## Introduction

The constitutive androstane receptor (CAR), a member of the nuclear receptor superfamily (subfamily 1, group I, member 3 [NR1i3]), plays an important role in coordinating cellular responses to the stimulation of both exogenous and endogenous chemicals by regulating the expression of its target genes (Qatanani and Moore, 2005; Stanley et al., 2006; Plant, 2007). Originally cloned as a constitutively activated receptor without a clearly defined biological function, the importance of CAR in xenobiotic metabolism was first appreciated when CAR was functionally linked to the long-known phenobarbital-mediated induction of hepatic cytochrome P450 (*CYP*) *2B* gene family (Honkakoski et al., 1998; Kawamoto et al., 1999). Encouraged by these findings, numerous investigations have been carried out to explore the role of CAR in xenobiotic metabolism, detoxification, and clearance (Maglich et al., 2002; Tolson and Wang, 2010). In humans, two functional enhancer modules, namely the phenobarbital-responsive enhancer module (PBREM) and the xenobiotic-responsive enhancer module (XREM), have been identified upstream of the *CYP2B6* gene and functionally characterized as the CAR binding sites in response to chemical stimuli (Honkakoski et al., 1998; Wang et al., 2003). CAR is also known to control the inductive expression of other CYP enzymes such as CYP3A4 (Goodwin et al., 2002), CYP2Cs (Ferguson et al., 2002; Gerbal-Chaloin et al., 2002), CYP2A6 (Wortham et al., 2007), and to a lesser extent CYP1A1 and CYP1A2 (Yoshinari et al., 2010), which contribute to the metabolism of approximately 75% of clinically used drugs and the detoxification of numerous environmental chemicals (Johansson and Ingelman-Sundberg, 2010). Further studies have extended CAR target genes including those encoding phase II enzymes such as the uridine diphosphate glucuronosyltransferase (UGT) isoforms (i.e., UGT1A1, UGT1A6, and UGT1A9) (Sugatani et al., 2005; Osabe et al., 2008; Buckley and Klaassen, 2009), glutathione S-transferases and sulfotransferases (Maglich et al., 2002; Yanagiba et al., 2009), as well as efflux and uptake drug transporters such as multidrug resistance-associated proteins (MRPs) (Cherrington et al., 2002, 2003), multidrug resistance protein 1 (MDR1) (Burk et al., 2005a, 2005b; Cerveny et al., 2007), and organic anion-transporting polypeptide 1 (OATP1) (Ding et al., 2006; Osabe et al., 2008). In addition to its broad spectrum of target genes, CAR also senses numerous xenobiotics and endobiotics as activators or deactivators and translates chemical stimulation into coordinated metabolism, detoxification, and clearance in the liver. Up-regulation of these drug-metabolizing enzymes or drug transporters by CAR activators may accelerate the biotransformation of co-administered drugs, usually leading to decreased therapeutic efficacy, enhanced toxicity, or increased bioactivation of prodrugs. For instance, recent studies in our lab have demonstrated that activation of CAR can enhance the bioactivation of cyclophosphamide (CPA) and facilitate CPA-based chemotherapeutic activity in leukemia cells (Wang et al., 2013). Understanding the role of CAR in mediating variable drug responsiveness and drug-drug interactions has become an intense focus of both academic and industrial research efforts and may lead to enhanced prediction of drug-drug interactions and xenobiotic-induced cytotoxicity.

Other than the well-established roles of CAR in the regulation of drug metabolism and transport, where it functions as a xenobiotic sensor, emerging evidence strongly suggests that CAR also modulates various hepatic functions that control diverse physiological and pathophysiological conditions, including energy metabolism, insulin signaling, cell proliferation, and tumor development (Fig. 1). In mice, selective activation of CAR significantly alleviated high fat diet-induced obesity and type 2 diabetics via a combined inhibition of lipogenesis, fatty acid synthesis, and gluconeogenesis, as well as the increase of energy expenditure in brown adipose tissues (Dong et al., 2009; Gao et al., 2009; Masuyama and Hiramatsu, 2012). Particularly, CAR influences energy homeostasis by suppressing the expression of phosphoenolpyruvate carboxykinase (PEPCK), glucose-6-phosphatase (G6Pase) (Kachaylo et al., 2012), sterol regulatory element-binding protein 1c (Roth et al., 2008), acetyl-CoA carboxylase 1, fatty acid synthase (FAS), and stearoyl-CoA desaturase-1 (SCD-1) (Du et al., 2008). The essential role of CAR in phenobarbital- and 1,4-bis[2-(3,5-dichloropyridyloxy)] benzene (TCPOBOP)-induced tumor promotion was initially established by using CAR knockout and wild-type mice (Yamamoto et al., 2004; Huang et al., 2005). In this regard, the known tumor promoters stimulated cancer progression by a CAR-dependent perturbation of the expression of the growth arrest and DNA damage-inducible 45 beta (GADD45B) (Columbano et al., 2005), the murine double minute 2 (mdm2) (Huang et al., 2005), as well as the newly identified tubulin alpha 8 (TUBA8) (Kamino et al., 2011a). In contrast to these observed roles of murine CAR in tumor development, activation of human (h) CAR by the selective activator, 6-(4-chlorophenyl)imidazo[2,1-b][1,3]thiazole-5-carbaldehydeO-(3,4-dichlorobenzyl)oxime (CITCO), appears to be associated with cell cycle arrest and enhanced apoptosis in human brain tumor stem cells, illustrating an anti-cancer potential (Chakraborty et al., 2011). Moreover, the enhanced cell proliferation by phenobarbital in the liver of wild-type mice was completely abrogated in the double-humanized CAR and pregnane X receptor (PXR) mouse model (Ross et al., 2010). Although the underlying mechanisms of the significant species differences of CAR in tumor development are largely unknown, such variances might be attributed to the divergent regulation of differential genes governing DNA synthesis, cell proliferation, apoptosis, and migration by hCAR vs. its rodent counterparts (Ross et al., 2010; Kamino et al., 2011b; Takizawa et al., 2011). Collectively, findings from these initial basic investigations hold the potential to advance CAR from a well-known xenobiotic sensor to an endobiotic modulator that may eventually become a promising drug target for metabolic disorders as well as cancer therapy.

**Figure 1:**
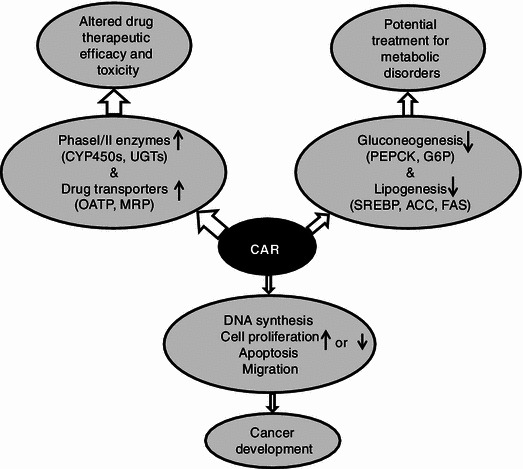
**Schematic illustration of biological functions of CAR**. The size of hollow arrows indicates the abundance of available evidence for each function of CAR. Up and down black arrows symbolize increased and decreased gene expression, respectively

Unlike PXR, the closest relative of CAR in the nuclear receptor superfamily tree, CAR is constitutively activated in nearly all immortalized cells and spontaneously accumulated in nuclei of these cells prior to chemical stimulated activation (Kawamoto et al., 1999). Moreover, CAR is featured as a nuclear receptor that could be transactivated through either the classical direct ligand binding or a mutedly defined ligand-independent indirect mechanism (Kawamoto et al., 1999; Maglich et al., 2003). These characteristics make the studies of CAR activation extremely challenging and pose major difficulties for evaluating drug-mediated CAR activation *in vitro*. This review is aimed to highlight the recent advances in our understanding of the molecular mechanisms behind drug-mediated nuclear translocation and activation of CAR, with a particular focus centered on signaling pathways that contribute to indirect activation of CAR.

## Activation of Car

As a so-called orphan receptor, CAR can be activated by a broad array of xenobiotic chemicals, often at micromolar concentrations, which differs from the classical steroid-hormone receptors which respond to endogenous ligands at nanomolar concentrations (Giguere, 1999; Tzameli and Moore, 2001). Structurally, however, CAR shares common functional features with other typical nuclear receptors, including a highly variable N-terminal AF1 domain, a DNA binding domain (DBD), a ligand-binding domain (LBD), and a C-terminal AF2 domain. The highly conserved DBD contains unique structures that can recognize and bind to specific promoter regions in target genes, namely xenobiotic response elements such as the aforementioned PBREM and XREM in the CYP2B6 promoter. Response elements binding to CAR are usually composed of two direct repeats of the consensus hexametric sequence of AG(G/T)TCA spaced by three to four nucleotides (i.e., DR3 or DR4) (Makinen et al., 2002). In the nucleus, CAR only binds to its response elements after forming heterodimers with the retinoid X receptor (RXR). The X-ray crystal structure of the hCAR/RXR LBDs reveals that CAR contains a single-turn Helix X that restricts the conformational freedom of the C-terminal AF2, and a relatively small ligand binding pocket (Xu et al., 2004). Such features permit CAR to interact with co-activator proteins and maintain a constitutively activated status once translocated into the nucleus. Importantly, although CAR shares several common characteristics with classical nuclear receptors, increasing evidence suggests that CAR can be activated by both direct ligand-binding and ligand-independent mechanisms (Kawamoto et al., 1999; Maglich et al., 2003). To this end, it appears that CAR activation is a multi-step process and most identified CAR activators may not directly bind to the receptor.

### Direct activation

Owing to the constitutive activation of CAR, the initial search for CAR ligands has resulted with the identification of androstenol and androstanol as inverse-agonists of CAR (Forman et al., 1998). Mechanistically, these androstanes convert CAR from constitutive to basal activity by disrupting the salt bridge that locks the H12 helix in its active conformation, promoting co-activator release from the LBD without interfering CAR/RXR dimerization or DNA binding (Shan et al., 2004). Subsequent studies uncovered TCPOBOP, the most potent known member of the phenobarbital-like class of CYP2B inducers, as the first agonist of mouse (m) CAR, in that TCPOBOP dose-dependently restores mCAR activity following inhibition by the inverse agonists (Tzameli et al., 2000). Notably, mutation of key residues inside the mCAR ligand-binding pocket entirely eliminated the stimulatory effect of TCPOBOP, as well as the inhibitory effect of androstanes, without affecting the constitutive activity of CAR (Tzameli et al., 2000). These results clearly establish CAR as a xenobiotic responsive modular protein that can be activated/deactivated by binding with agonistic and antagonistic ligands, respectively.

Importantly, CAR exhibits remarkable species selectivity in its ligand binding and activation profiles, which makes direct extrapolation of findings from mouse to human extremely risky. For example, TCPOBOP and estradiol activate mouse but not human CAR, and pharmacological concentrations of androstanol, progesterone, and testosterone repress mouse but not human CAR (Handschin and Meyer, 2003; Maglich et al., 2003). The first selective hCAR agonist, the imidazothiazole derivative-CITCO, came through a combination of *in vitro* and cell-based screening in 2003 (Maglich et al., 2003). CITCO selectively binds to hCAR and activates CAR target genes in human primary hepatocytes (Maglich et al., 2003; Ferguson et al., 2005; Faucette et al., 2006). Recent evidence also reveals that CITCO can efficiently enhance recruitment of co-activators to the LBD of hCAR by competing with antagonists such as PK11195 (Li et al., 2008) and metformin (Yang et al., 2013). However, CITCO only moderately enhances the constitutively activated hCAR (less than 2-fold), compared with that of TCPOBOP for mCAR (5- to 10-fold) in cell-based luciferase reporter assays (Tzameli et al., 2000; Maglich et al., 2003). Moreover, CITCO also activates human PXR and induces PXR target genes at higher concentrations, leaving direct comparison of human CAR and PXR target genes yet challenging (Maglich et al., 2003). To date, there is no pure hCAR agonist reported. Other chemicals exhibiting agonistic effects on hCAR such as the antimalarial artemisinin, the psychoactive diazepam and the anti-fungal myclobutani, are also associated with potent activation of hPXR (Burk et al., 2005b; Li et al., 2009). Overall, ligand-dependent direct activation of CAR may still rely on its initial step of translocating CAR into the nucleus. Once inside the nucleus, CITCO bound hCAR adopts a conformation similar to the constitutively active apo-CAR and maintains the intrinsically high consititutive activity.

### Indirect activation

The hallmark feature that differentiates CAR from classical nuclear receptors lies in its ligand-independent nuclear accumulation and constitutive activation once expressed inside the nucleus of cells. To date, numerous CAR activators have been identified, including clinically used drugs, environmental chemicals, and endogenous steroid metabolites (Qatanani and Moore, 2005; Li and Wang, 2010; Molnar et al., 2013). Most of these activators however do not bind directly to CAR; instead activating CAR by stimulating its nuclear translocation in a ligand-independent manner (Li et al., 2009). For example, the typical CYP2B inducer and CAR activator phenobarbital does not bind directly to CAR but induces CAR transcriptional activition exclusively via nuclear translocation (Kawamoto et al., 1999; Moore et al., 2000). Notably, constitutive activation of CAR is not always a beneficial feature. In this regard, CAR activation can enhance the metabolism and toxicity of some drugs, such as acetaminophen (Zhang et al., 2002), and potentially increase tumor propensity by stimulating cell proliferation (Takizawa et al., 2011). To accommodate such potential adversity, CAR is primarily located in the cytoplasm prior to activation in primary hepatocytes and intact liver *in vivo* (Kawamoto et al., 1999; Li et al., 2009). In this native hepatocyte environment, CAR is spontaneously sequestered in the cytoplasm as a multi-protein complex including the heat shock protein 90 (Hsp90), cytoplasmic CAR retention protein, protein phosphatase 1 regulatory subunit 16A, and potentially other yet unidentified proteins (Kobayashi et al., 2003; Yoshinari et al., 2003; Sueyoshi et al., 2008). Upon the stimulation of phenobarbital-type indirect activators or CITCO/TCPOBOP-like direct ligand-binding, CAR disassociates from the cytoplasmic localized protein complex and moves into the nucleus. It was believed that this process is regulated by protein kinase-mediated phosphorylation/dephosphorylation of CAR. A major breakthrough came with the identification of the conserved threonine (Thr)-38 of human CAR as the primary residue that governs nuclear translocation and activation of CAR (Mutoh et al., 2009). Dephosphorylation of the Thr-38 appears to be essential for CAR translocation regardless of exposure to direct or indirect activators (Mutoh et al., 2009). The exact molecular mechanisms controlling Thr-38 CAR phosphorylation/dephosphorylation remain to be fully understood. However, several kinase signaling pathways have recently been suggested to be important in the phosphorylation of CAR.

### Protein phosphatase 2A (PP2A)

The role of protein kinase-based signaling pathways in controlling phenobarbital-mediated induction of CYP450s had been proposed, even before CAR was recognized as the fundamental target of phenobarbital. Early studies showed that both activation of protein kinase A (PKA) by elevated intracellular cyclic adenosine monophosphate (cAMP) and the inhibition of protein phosphatases PP1 and PP2A by okadaic acid (OA) resulted in complete repression of phenobarbital-inducible *CYP* gene transactivation in primary rat hepatocytes (Sidhu and Omiecinski, 1995, 1997). Although the transcription factor(s) that drive the phenobarbital induction event was/were yet to be determined, these results indicated that both PKA and protein phosphatase pathways exert marked roles in modulating the signaling of phenobarbital-mediated CYP induction. After establishing CAR as the critical DNA-binding protein required for phenobarbital response, Negishi and colleagues demonstrated that OA pretreatment was sufficient to inhibit phenobarbital-mediated nuclear translocation of CAR and induction of Cyp2b10 in primary mouse hepatocytes, suggesting that CAR nuclear accumulation is most likely regulated by a dephosphorylation-sensitive signaling cascade (Kawamoto et al., 1999). Further studies from the same research group revealed that CAR exists as a complex with Hsp90 in the cytoplasm of non-induced mouse liver hepatocytes. More importantly, phenobarbital treatment recruited PP2A to the protein complex, which led to the dephosphorylation of CAR (Yoshinari et al., 2003).

Realizing the importance of phosphorylation/dephosphorylation in CAR nuclear translocation and activation, the next significant question to be answered was which amino acid residue(s) is/are responsible for such chemical-stimulated signaling. Serial-deletion and site-directed mutagenesis of CAR led to the identification of a leucine-rich motif (LXXLXXL) close to the C-terminal region, namely the xenobiotic response sequence (XRS), as the potential functional unit which dictates the nuclear translocation of CAR in response to various phenobarbital-type inducers (Zelko et al., 2001; Xia and Kemper, 2007). Nevertheless, these residues were not direct targets of either PP2A or PKA. A real breakthrough in this regard came with the diligent work by Mutoh et al. in 2009, in which the Thr-38 residue of hCAR was established as the primary determinant for chemical-mediated phosphorylation/ dephosphorylation of CAR, while dephosphorylation of Thr-38 is a prerequisite for CAR translocation into the nucleus (Mutoh et al., 2009). Consistent with earlier observations, treatment with OA increased the phosphorylation of CAR at Thr-38 and sequestered CAR in the cytoplasm of mouse primary hepatocytes (Mutoh et al., 2013).

### Extracellular signal-regulated kinase (ERK) and p38 mitogen-activated protein kinase (MAPK)

Accumulating evidence has demonstrated that expression of various CYP enzymes was significantly repressed during liver regeneration, infection or inflammation, suggesting cellular signaling molecules such as growth hormones and cytokines may play a role in the expression of xenobiotic-metabolizing CYPs (Bauer et al., 2004; Koike et al., 2007). In particular, two independent studies provided strong evidence to show that phenobarbital-dependent activation of the rat CYP2B1 promoter was repressed by the presence of epidermal growth factor (EGF) but promoted by U0126, a known inhibitor of the MEK-ERK signaling pathway (Bauer et al., 2004; Joannard et al., 2006). Encouraged by these observations, Negishi and coworkers provided further mechanistic evidence suggesting ERK is an endogenous signal, regulating CAR phosphorylation and nuclear translocation, by which U0126-mediated Cyp2b10 induction via ERK1/2 deactivation was completely abrogated in CAR knockout mice (Koike et al., 2007). Moreover, co-immunoprecipitation experiments revealed that activated ERK1/2 co-precipitated only with the Thr-38 phosphorylated CAR, where the C-terminal located XRS appears to be essential for this interaction (Osabe and Negishi, 2011). This interaction was significantly increased after EGF exposure while treatment with U0126 decreased the level of CAR phosphorylation at Thr-38 and eventually released CAR into the nucleus (Osabe and Negishi, 2011).

An outstanding phenomenon observed was that ectopic expression of hCAR in HepG2 cells does not convey optimal induction of CYP2B6 compared to what was observed in human primary hepatocytes; many other cellular signals have been shown to regulate the activation of CAR. Recently, the p38 MAPK was identified as a required factor optimizing CAR activation and CYP2B6 induction in liver cells (Saito et al., 2013). In human primary hepatocytes, p38 MAPK is highly activated, which significantly differs from that in human hepatoma cell lines, including HepG2 cells. Activation of p38 MAPK by anisomycin robustly potentiated induction of CYP2B6 mRNA by CAR activators in HepG2 cells to levels that were comparable to what was observed in ligand-treated human primary hepatocytes. The potential significance of p38 MAPK in chemical-elicited CAR activation was also indirectly supported by the facts that phenobarbital-mediated induction of CYP2B1 was stimulated in the liver of diabetic rats where p38 MAPK was activated by the disease itself (Yoshida et al., 1996); while CYP induction by phenobarbital was attenuated in tumor-bearing rats where p38 MAPK was down-regulated (Numazawa et al., 2005). Nonetheless, a definite role of p38 MAPK in CAR activation has yet to be established, given that activation of p38 MAPK appears to enhance some but not all target genes of hCAR.

### Epidermal growth factor receptor (EGFR)

Previous studies have shown that phenobarbital-induced *CYP2B* gene transactivation could be effectively repressed by growth factors, such as EGF and insulin-like growth factor (IGF) (Bauer et al., 2004; Kietzmann et al., 1999; Thasler et al., 2006). EGFR is a member of the ErbB family of receptors that coordinates extracellular signals, such as EGF, to cellular signaling cascades and eventually promotes cell proliferation (Di Fiore et al., 1990). Recent studies by Mutoh et al., identified EGFR as a phenobarbital-responsive receptor that mediates CAR dephosphorylation and activation in mouse primary hepatocytes (Mutoh et al., 2013). As shown in this study, phenobarbital antagonizes EGF-stimulated EGFR phosphorylation and activation; abrogation of EGFR signaling further induces the dephosphorylation of the downstream receptor for activated C kinase 1 (RACK1) at the residue of Tyr-52. The dephosphorylated RACK1 then directly recruits PP2A to the cytosol localized CAR protein complex, where it dephosphorylates and releases CAR into the nucleus (Fig. 2). More importantly, this study provides the first evidence that phenobarbital can directly bind to EGFR at pharmacologically relevant concentrations. Given that phenobarbital is often referred to as an “orphan compound” without a known direct target, EGFR may represent one of the molecular targets that initiates phenobarbital-mediated cellular responses, including CAR activation. On the other hand, phenobarbital may not function as a prototypical EGFR inhibitor, such as gefitinib and erlotinib, which can antagonize EGFR-mediated cell proliferation and tumor development (Nakajima et al., 2012; Shin et al., 2013). In fact, phenobarbital itself is a potent tumor promoter in rodent animals via a CAR-dependent mechanism (Huang et al., 2005; Yamamoto et al., 2004). Therefore, it is reasonable to speculate that phenobarbital might be an atypical antagonist of EGFR, which only selectively inhibits certain downstream events of EGFR signaling.

**Figure 2:**
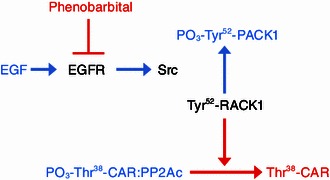
**Antagonistic effect of phenobarbital on EGFR signaling and CAR activation**. Arrows indicate activation and the blunt arrow represents deactivation. (This figure was adopted from Mutoh et al., 2013, Science Signaling)

### AMP activated protein kinase (AMPK)

AMPK is an enzyme that functions as an energy sensor by regulating cellular energy metabolism and homeostasis. AMPK plays an important role in fatty acid oxidation, glucose uptake, and hepatic lipogenesis by reacting to the fluctuation of the cellular AMP:ATP ratio (Hardie et al., 2012; Inoki et al., 2012). Recent studies suggested that AMPK is involved in CAR-regulated *CYP2B* gene induction by phenobarbital-type inducers, but the precise role of AMPK in the activation of CAR remains controversial. Studies from Meyer and colleagues showed that AMPK activator 5-AMINO-1-β-Dffff-ribofuranosyl-imidazole-4-carboxamide (AICAR), or expression of a constitutively active form of AMPK, mimicked phenobarbital-mediated induction of CYP2B6 in hepatoma cell lines (Rencurel et al., 2005). On the other hand, liver-specific deletion of AMPK catalytic subunits in mice impaired the inductive expression of Cyp2b10 and Cyp3a11, but did not inhibit the nuclear accumulation of CAR induced by phenobarbital (Rencurel et al., 2006). Therefore, the authors presumed the existence of another control step of CAR signaling independent of translocation. However, an *in vivo* study conversely showed that AICAR and metformin induced CAR nuclear translocation but failed to induce hepatic *CYP2B* genes in mice and rats, suggesting AMPK activation is not sufficient for CYP2B induction (Shindo et al., 2007).

In another study, AICAR was shown to prevent nuclear translocation of CAR and repress phenobarbital-induced CYP2B expression in rat primary hepatocytes (Kanno et al., 2010). In the same study, metformin and the constitutively active form of AMPK, however, enhanced PBREM-driven transactivation by phenobarbital, suggesting AICAR inhibits CAR translocation in an AMPK-independent manner. Most recently, we have shown that metformin dramatically repressed phenobarbital/CITCO-induced CYP2B6 expression through inhibiting dephosphorylation and nuclear translocation of CAR (Yang et al., 2013). Consistent with this observation, our data also demonstrated that AICAR mimicked the effect of metformin on CYP2B6 suppression, and such repression was partially but concentration-dependently restored by co-treatment with compound C (6-[4-(2-piperidin-1-yl-ethoxy)-phenyl)]-3-pyridin-4-yl-pyrrazolo[1,5-a]-pyrimidine, a known inhibitor of AMPK. Although sequence alignments of the conserved Thr-38 region of CAR revealed no consensus AMPK site, signaling molecules downstream of the AMPK pathway such as PKC (He et al., 2009) may function as the switch controlling CAR phosphorylation and its disassociation from the retaining protein complex.

Collectively, these studies implicate rather contradictory outcomes when connecting CYP2B transactivation and CAR nuclear translocation to AMPK activation. Some of the disputes however, can be explained at least in part by the diverse physiological properties of different species or cell systems used in these studies, such as immortalized cell lines vs. primary hepatocytes, human cells vs. rodent cells, and *in vivo* vs. *in vitro*. In addition, energy status and nutritional environment of the cells can also influence phenobarbital regulation of the *CYP2B* gene (Yoshida et al., 1996; Rencurel et al., 2006).

### Transcriptional regulation of CAR

Although the biological function of CAR relies predominantly on chemical-mediated activation/deactivation through direct or indirect mechanisms, the expression level of CAR in response to endogenous signals or xenobiotic chemicals may also influence the downstream regulation of its target genes. It is well known that dramatic interindividual differences exist in the expression of hepatic CYP2B6, the prototypical target gene of hCAR (Wang and Tompkins, 2008). Nevertheless, the molecular mechanism(s) underlying this large variability remains elusive. In comparison of a panel of 12 individual human liver samples, Chang et al. revealed that substantial interindividual differences of hCAR expression in these samples were significantly and positively correlated with that of CYP2B6, indicating the abundance of this transcription factor may contribute to the varied expression of the *CYP2B6* gene in human liver (Chang et al., 2003). Other studies highlighted that different from cognate CAR activation, expression of CAR can be induced by a number of xenobiotics including the glucocorticoid receptor agonist (dexamethasone) (Pascussi et al., 2000) and peroxisome proliferator-activated receptor (PPAR)-alpha agonists (WY14643 and ciprofibrate) (Saito et al., 2010). *In silico* analysis of the human CAR 5′-regulatory region led to the identification of a putative glucocorticoid responsive element located between -4477 and -4410 base pair (Pascussi et al., 2003), a functional PPAR-alpha responsive element around -4400 base pair, as well as a conserved hepatocyte nuclear factor 4 alpha (HNF4α) binding site from upstream of the transcriptional start site (Ding et al., 2006).

Previous animal studies have demonstrated that fasting and caloric restriction increase the expression and activity of CAR which in turn coordinates an adaptive response by slowing down the energy expenditure. CAR knockout animals were unable to couple the metabolic adjustment and lost more weight (Maglich et al., 2004; Qatanani et al., 2005). Given that fasting typically increases the plasma level of free fatty acids that are natural ligands of PPARα, and elevated interaction between PGC-1α and HNF4α is a hallmark of fasting adaptation, functionally establishing CAR as a target gene of PPARα and HNF4α provides a novel mechanistic model for CAR in energy homeostasis (Ding et al., 2006). Additional evidence indicated that stress-activated protein kinase and ERK signaling pathways are also associated with altered expression of CAR under serum-starvation stress (Osabe et al., 2009). Most recently, our own data have unexpectedly revealed that an insulin-like growth factor-1 receptor (IGF-1R) inhibitor (BMS-665351) significantly induced the expression of CYP3A4 in human primary hepatocytes without activation of either CAR or PXR, instead it selectively induced the expression of CAR (Li et al., 2012). Intriguingly, BMS-665351 did not activate either glucocorticoid receptor or PPARα at concentrations that induced the expression of CYP3A4 and CAR, implying additional, yet unknown mechanisms may be involved in the transcriptional regulation of CAR. Collectively, in comparison with the heightened focus on the activation and deactivation of CAR, much less is known regarding how the expression of CAR itself is controlled under the challenge of both endogenous and xenobiotic chemicals. Clearly, transcriptional regulation of the regulator would represent another layer of CAR biology.

## Concluding Remarks

It is evident now that CAR has evolved into a sensor of both xenobiotic and endobiotic chemicals by governing the transcription of genes associated with drug metabolism and transport, energy homeostasis, and cell proliferation. Our understanding of the role of CAR in gene regulation as well as the mechanisms of its activation has increased remarkably during the past 15 years. As summarized in this review, an astonishing number of cellular factors and foreign compounds intertwine in the regulation of CAR biological functions. Although CAR shares several common features with its sister receptor PXR, where they overlap in a number of target genes and xenobiotic activators, the mechanisms of CAR activation have been proven to be relatively unique. To date, mounting evidence demonstrates that CAR can be activated through both classical ligand binding and ligand-independent mechanisms, with indirect activation appearing to be predominant. Seminal works by Negishi and colleagues have shown that the phosphorylation status of CAR is pivotal for its cellular localization and activation, which could be influenced by many protein kinase signals (Mutoh et al., 2009). In the meantime, the cellular expression of CAR itself appears to be affected by certain signaling molecules. Together, these data indicate that CAR may represent a cell signaling-regulated nuclear receptor rather than a typical ligand-dependent nuclear receptor (Fig. 3). Given that CAR can be activated both directly and indirectly, it is essential to keep in mind that the ligand binding and kinase signaling may interconnect to achieve the optimal activation of this receptor. Undoubtedly, better understanding of the signaling control of CAR activation will eventually benefit the prediction of metabolism-based drug exposure as well as the development of CAR modulators as potential drug candidates.

**Figure 3:**
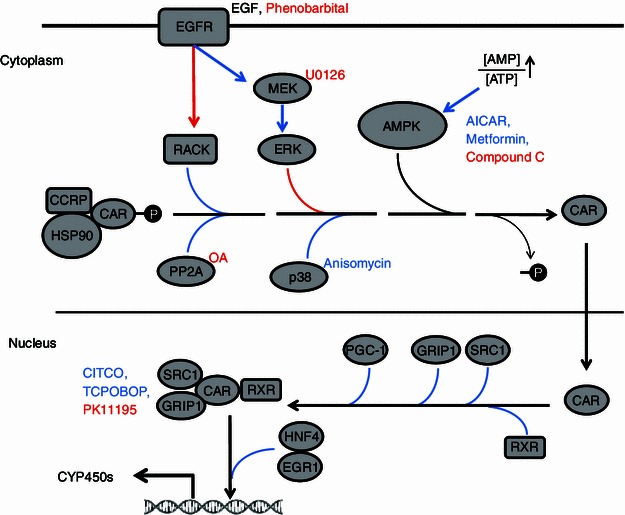
**Signaling control of CAR activation**. Chemicals illustrating activation or deactivation of a signaling pathway are denoted in blue and red, respectively

## Abbreviations

AICAR: 5-aminoimidazole-4-carboxamide-1-β-D-ribofuranoside
AMPK: AMP activated protein kinase
cAMP: cyclic andenosine monophosphate
CAR: constitutive androstane receptor
CITCO: 6-(4-chlorophenyl)imidazo[2,1-b][1,3]thiazole-5-carbaldehydeO-(3,4-dichlorobenzyl)oxime
EGF: epidermal growth factor
EGFR: EGF receptor
EGR1: early growth response 1
ERK: extracellular signal regulated kinase
GRIP1: glucocorticoid receptor-interacting protein 1
HNF4α: hepatocyte-enriched nuclear receptor 4 alpha
HSP90: heat shock protein 90
MAPK: mitogen-activated protein kinase
OA: okadaic acid
PBREM: phenobarbital-responsive enhancer module
PGC-1α: peroxisome proliferator-activated receptor gamma coactivator-1 alpha
PKA: protein kinase A
PP2A: protein phosphatases 2A
PXR: pregnane X receptor
RACK1: receptor for activated C kinase 1
RXR: retinoid X receptor
SRC1: steroid receptor co-activator 1
TCPOBOP: 1,4-bis[2-(3,5-dichloropyridyloxy)] benzene
UGT: uridine diphosphate glucuronosyltransferase
XREM: xenobiotic-responsive enhancer module
